# Microbial Diversity and Nutritional Properties of Persian “Yellow Curd” (*Kashk zard*), a Promising Functional Fermented Food

**DOI:** 10.3390/microorganisms8111658

**Published:** 2020-10-26

**Authors:** Shadi Pakroo, Armin Tarrah, Vinícius da Silva Duarte, Viviana Corich, Alessio Giacomini

**Affiliations:** Department of Agronomy Food Natural Resources Animal and Environment (DAFNAE), University of Padova, Viale dell’Università 16, 35020 Legnaro, PD, Italy; shadi.pakroo@gmail.com (S.P.); tarrah.armin@gmail.com (A.T.); vs_duarte01@hotmail.com (V.d.S.D.); alessio.giacomini@unipd.it (A.G.)

**Keywords:** Iran, traditional food, *Kashk zard*, 16S rRNA analysis, nutrients

## Abstract

“Yellow curd” (YC) is one of the most popular homemade Persian fermented foods and is consumed by many people. Notwithstanding, no studies are available to date on its nutritional and microbiological composition. In this study, we examined YC samples obtained from different local markets of Sistan and Baluchestan province, Iran. The results of the chemical analyses revealed a homogenous content of protein (13.71% ± 1.07), lipids (4.09% ± 0.73), and carbohydrates (61% ± 2.13) among the samples. By comparing the average mineral content of YC with yogurt, many relevant differences were detected. Apart from the calcium content, which was similar on average to that of YC, all other minerals tested are present in higher amounts in YC than in yogurt. The analysis of the main sugars present (i.e., lactose, galactose and glucose) highlighted relevant differences among samples, indicating that different YC samples contain natural strains with different capabilities to metabolize sugars. The concentration of galactose in YC samples should be taken into consideration by galactose intolerant people. From the microbiological perspective, the metagenomics analysis revealed that lactic acid bacteria, and particularly the genera *Lactobacillus*, *Pediococcus*, and *Streptococcus*, were dominant in YC. The information provided shows that YC is an interesting base for the preparation of novel functional foods with a good content of beneficial bacteria.

## 1. Introduction

Iran is a country rich in traditional foods that come from an ancient food preparation history. “Yellow curd” (YC) is one of the most popular homemade food products in the south-eastern part of Iran (i.e., Sistan and Baluchestan provinces), but it is also largely produced and consumed in other parts of the country, including the Khorasan province and the capital, Tehran.

Generally, YC is prepared by a combination of homemade yogurt (made from cow milk), wheat flour, different types of local aromatic herbs and spices (dill, coriander, cumin, turmeric, garlic) and salt. The preparation process includes two fermentation steps. First, a dough is made by adding wheat flour to the yogurt and maintaining the resulting mixture inside a specific earthen container for two weeks in a dark and warm (around 30 °C) place. Then, fresh yogurt and seasonings are added, kneaded with the dough and the resulting mix is again stored at the same conditions for another 7 to 10 days, during which a second fermentation takes place. The product is then distributed outside on the surface of sheets of textile and left to dry for up to 10 days. Lastly, the dried YC is ground to get a granulated product of 1–3 mm sized particles. YC is consumed as a soup, prepared by dissolving about 6 g of powder in 1 L of water. To date, no studies are available in the literature on the chemical and microbiological characteristics of this product. Indeed, information about YC composition would be important to determine its nutritional value and the possible influence of food components on microbial selection and development. Knowledge of its microbiota composition would be relevant, since it is ascertained that microbes considerably influence many characteristics of fermented products, including safety, sensory characteristics, and nutritional aspects. To our knowledge, this study is the first report on the microbiological and chemical characteristics of YC, a poorly studied traditional product which constitutes one of the most consumed daily meals by people in several regions of Iran.

## 2. Materials and Methods

### 2.1. Sample Collection and Processing

**Figure 1:**
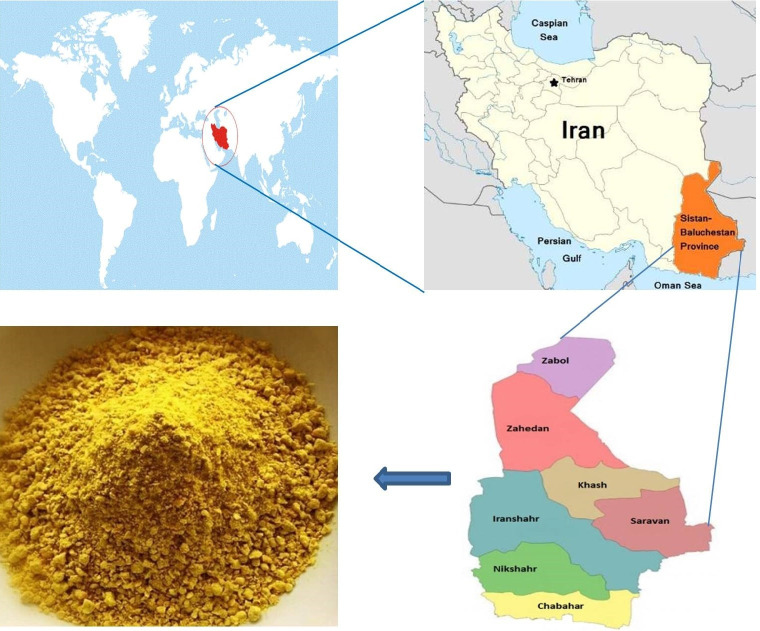
Geographic location of the Sistan and Baluchistan provinces in Iran and image of “yellow curd” (YC) powder.

A total of 13 “yellow curd” (YC) samples were collected from seven different local household traditional markets in the Sistan and Baluchestan provinces (from different cities) in the south-east of Iran during August 2018 (Figure 1). Samples (approximately 100 g each) were collected in sterile plastic tubes, which were tightly capped and stored at room temperature. Samples were named from IRZ1 to IRZ13.

### 2.2. pH Measurement

pH determination of YC samples was performed according to Zaika et al. [1] with slight modification. Briefly, 20 g of YC samples were added to 160 mL of distilled water and mixed thoroughly. The solution was filtered, and 50 mL of this solution was further diluted 1:1 with 50 mL of distilled water. The pH was measured using a digital pH meter (Orion Star A211, ThermoFisher Scientific, Waltham, MA, USA) by immersing the electrode into the solution.

### 2.3. 16S rRNA Gene Amplicon Target Sequencing

Total genomic DNA was extracted using the DNeasy PowerSoil Microbial Kit (Qiagen, Valencia, CA, USA) according to the manufacturer’s instructions. The quality and quantity of the extracted DNA were assessed by 1% agarose gel electrophoresis and with a Spark 10M spectrophotometer (Tecan Trading AG, Männedorf, Switzerland), respectively. The V3–V5 regions of the 16S rRNA genes were amplified by PCR and sequenced using an Illumina MiSeq desktop sequencer (Eurofins Genomics Germany GmbH, Ebersberg, Germany), producing 300 bp paired-end (PE) reads. Three separate aliquots (technical replicates) were analyzed for each YC sample. Then, 16S rRNA sequences were analyzed using the software CLC Genomics Workbench (V.8.0.2) with the microbial genomics module plugin (QIAGEN Bioinformatics, Germany). Finally, the Operational Taxonomic Units (OTUs) that were not automatically attributed were manually assigned to the genus level by similarity, using BLASTN Megablast. Raw reads were deposited in the Sequence Read Archive (SRA) database (10.1093/nar/gkq1019). SRA accession numbers are reported in Spreadsheet 1.

### 2.4. Chemical and Mineral Compositions of YC Samples

Grinding of YC samples was done using a GRINDOMIX GM200 Retsch homogenizer for 10 s at 4500 rpm. Protein and lipid content, as well as ash and dry matter weights, were determined as described by William [2]. Minerals (namely calcium (Ca), potassium (K), magnesium (Mg), manganese (Mn), sodium (Na), phosphorus (P), and zinc (Zn)) were measured as described by [3], using a Milestone Start microwave oven (Milestone Srl Sorisole Bergamo, Italy) and the results were collected using a Inductively Coupled Plasma Optical Emission Spectrometry (ICP-OES) Spectro Arcos (Spectro Analytical Instruments GmbH, Kleve, Germany). Each analysis was performed in triplicate.

### 2.5. Quantification of Sugar Content

Lactose, glucose, and galactose quantification in all samples was performed in triplicate by high-performance liquid chromatography (HPLC). All solvents were filtered using 0.45 μm filters. Ultrapure water was used for all dilutions and to prepare lactose, glucose and galactose standard solutions at concentrations between 0.005% (0.05 g/L) and 0.25% (2.5 g/L). Briefly, 5 g of YC samples were mixed with 50 mL of 7% perchloric acid (HClO_4_) in a volumetric flask and agitated for 10 min to precipitate proteins. Then, the pH was adjusted to 7.0 with 0.1 N NaOH and the solution was centrifuged at 6000 rpm for 2 min. Then, 1 mL of each sample was filtered using 0.22 μm Polytetrafluoroethylene (PTFE) filters. Aliquots of 20 μL were then injected into the HPLC for the analysis using a HyperRez XP Carbohydrate Ca^2+^ (300 × 7.7 mm) column and the following parameters: mobile phase = 100% ultrapure water, flow rate = 0.6 mL/min, pressure = 1100 psi, oven temperature = 80 °C, detection = RI (Refractive Index). The HPLC analysis time was 8:8 (min:s) for lactose, 10:4 (min:s) for glucose, and 11:25 (min:s) for galactose, respectively.

### 2.6. Statistical Analysis

Data collected from chemical and mineral analyses were evaluated by one-way analysis of variance (ANOVA) using Minitab software (version 19, Minitab software, State College, PA, USA).

The relative abundance of each OTU for every YC sample obtained from the bioinformatics analyses was normalized by log transformation log10 (x_i_ + 1). Bacterial similarity among samples was evaluated by ANalysis of SIMilarity (ANOSIM) with 9999 permutations using the Bray–Curtis similarity index in the PAleontological STatistics (PAST) 3.26 [4] software. The Statistical Analysis of Metagenomics Profiles (STAMP) software [5] was used to plot principal component (PC) diagrams, as well as to identify significant differences in terms of genus among different samples using ANOVA. Tukey–Kramer (*p* < 0.05) was chosen as the post-hoc test and Bonferroni was used for multiple test corrections.

Alpha diversity indexes were compared within all YC samples using ANOVA in GraphPad Prism 7 (GraphPad Software LLC, La Jolla, CA, USA). Tukey test (*p*  <  0.05) was used to adjust for multiple comparisons.

## 3. Results and Discussion

### 3.1. 16S rRNA Gene Amplicon Target Sequencing

In this study, we analyzed 12 samples of YC with three technical replicates each. By conducting a high throughput amplicon sequencing of the hypervariable regions V3–V5 of the 16S rRNA gene, a total of 943,135 high-quality sequences with an average length of 254 bp were obtained after the removal of low quality and chimeric sequences. Unfortunately, due to the excessively low number (less than 1000) of reads obtained, sample IRZ1 was excluded from the analyses, which then included 12 samples (from IRZ2 to IRZ13). The procedure adopted for DNA extraction did not reasonably determine the lysis of bacterial endospores; therefore, such microbes are not considered in the genetic analysis.

Regarding the analysis of alpha diversity, the rarefaction curves (an average number of sequences and biodiversity indices associated with each sample (Figure S1)) show that the sequencing depth was enough to cover the microbial diversity in most samples analyzed. Indeed, Good’s coverage index mean was 80% ± 11% (Spreadsheet 1), showing that most bacterial phylotypes were sampled and that YC biodiversity is described in an adequate manner, although an increasing depth of sequencing could have allowed the possible identification of some other less abundant taxonomic units.

**Figure 2:**
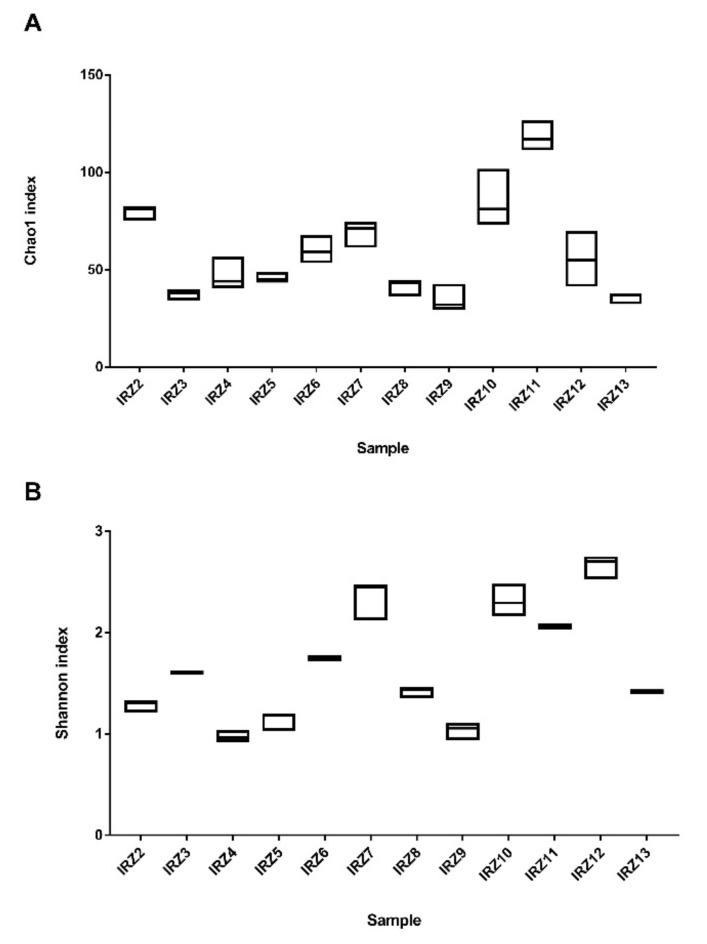
Alpha diversity: Chao1 (**A**) and Shannon (**B**) analyses regarding the number of species (richness) and microbial distribution (evenness), respectively, across all samples (*p* < 0.05).

The Chao1 and Shannon indices, accounting for the number of different species (richness) and microbial distribution (evenness), respectively, varied significantly among the samples (*p* < 0.05) (Figure 2). Indeed, multivariate analysis (ANOSIM, *p* = 0.0001, ANOSIM statistic R (R) = 0.9976) indicates a highly dissimilar microbial composition among YC samples (Figure S2), showing a relevant local influence on the bacterial diversity of these homemade products. Overall, principal components 1, 2 and 3 explain more than 93% of the variability (Figure S3).

**Figure 3:**
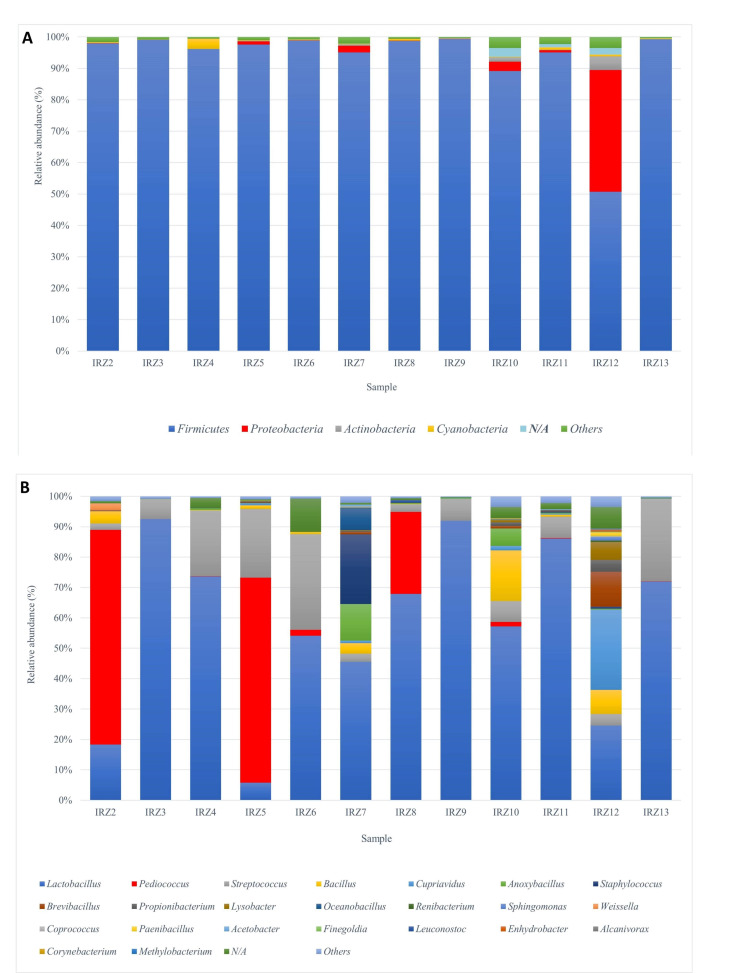
Relative abundance of bacterial groups of YC samples at the phylum (**A**) and genus (**B**) level.

After the clustering process (at a 97% similarity threshold), a total of 327 OTUs were identified. In total, 71 OTUs with more than 0.5% relative abundance were considered as “the most abundant”. In regard to taxonomy, it was possible to assign 76% of the sequences at the phylum level, 59% at the genus level, and 16% at the species level. At the phylum level, *Firmicutes* (92%) were highly predominant, followed by *Proteobacteria* (4%) (Figure 3A). This is in accordance with published studies [6,7] that reported that the bacterial microbiota of naturally fermented milk includes *Firmicutes*, *Proteobacteria*, *Actinobacteria*, and *Bacteroidetes*, but *Firmicutes* represent the large majority. Interestingly, the phylum *Bacteroidetes* was not included among the most abundant OTUs, probably as a consequence of the high sensitivity of these bacteria to oxygen present during YC production and storage [8]; it could also be considered an index of good hygienic environmental level, since *Bacteroidetes* are mainly bacteria of intestinal origin. Additionally, the significant presence of *Cyanobacteria* could reasonably be attributed to chloroplast DNA from plant material (i.e., vegetables and seasonings) that are added to the curd.

At the genus level, *Lactobacillus* (57%), *Pediococcus* (14%) and *Streptococcus* (12%) were the most abundant (Figure 3B; Spreadsheet 1). Members of the genus *Lactobacillus* have a relevant role in the production of traditional fermented foods, since this heterogeneous genus includes thermophilic species able to grow well by fermenting several sugars [9,10]. Of the bacterial sequences assigned to *Lactobacillus* species (*L. brevis*, *L. plantarum*, *L. reuteri*, *L. vaginalis*, and *L. zeae*), *L. plantarum* was identified in all samples in this study (Spreadsheet 1). As reported by several studies, this species is frequently isolated from traditional dairy products and contains strains with remarkable probiotic and technological properties [11,12]. We also conducted a MegaBLAST search to find the best hit at species level for the OTUs assigned to the genus *Lactobacillus*. Interestingly, this analysis allowed for the identification of three other species, namely *L. delbrueckii*, *L. pontis* and *L. fermentum*, which are frequently isolated from sourdoughs (naturally fermented flour–water mixtures used in the production of baked goods). These microbes could come from cereals that are added to YC in the manufacturing process, which is a potential source of lactobacilli [13].

**Figure 4:**
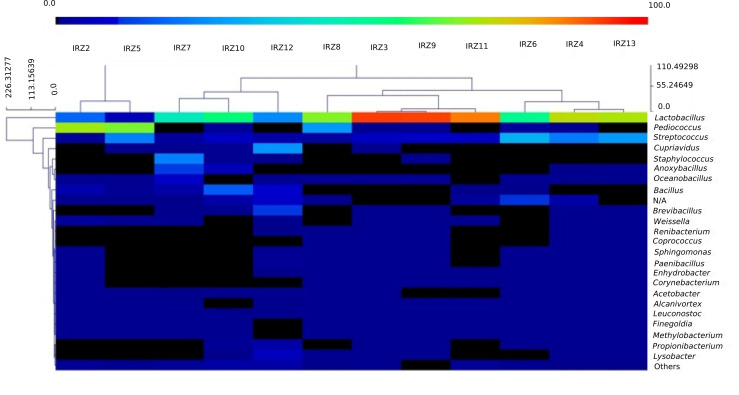
Heatmap reporting the levels of microbial diversity in terms of genera among YC samples.

A heat map of relative abundance and distribution based on bacterial genera (Figure 4) was constructed with the software MultiExperiment Viewer (MeV) [14]. A high presence of *Pediococcus acidilactici*, a species frequently found in traditional cereal fermentations but not typical of dairy products [15], was observed among the samples and found to be particularly present in three samples (IRZ2, IRZ5, and IRZ8). Interestingly, in these samples, a concomitant lower abundance of *Lactobacillus* was noticed that could be correlated to some antagonistic activity, possibly linked to the synthesis of antibacterial proteins (bacteriocins), a characteristic quite widespread among pediococci [16]. Indeed, *P. acidilactici* is a well-known bacteriocin (pediocins)-producing species active against Gram-positive bacteria (mainly *Listeria* and some Lactic Acid Bacteria (LAB)) that is of interest in the food industry for its potential application in biopreservation [15,17]. Furthermore, *P. acidilactici* has attracted attention as a promising probiotic, as evidenced by some studies [18,19]. The genus *Streptococcus* was found consistently in all samples at percentages ranging from 2% (IRZ2) to 31% (IRZ6) of relative abundance. Although 16S rRNA analysis used in this study cannot achieve the species level for this genus, a MegaBLAST search revealed 100% identity with *S. thermophilus* strains, including the type strain ATCC 19258. *S. thermophilus* is naturally present and industrially used worldwide in the manufacture of many homemade and industrial fermented dairy products such as yogurt, *tarag*, *kurut* and *dahi* [6,20].

### 3.2. Chemical Properties of YC Samples

**Figure 5:**
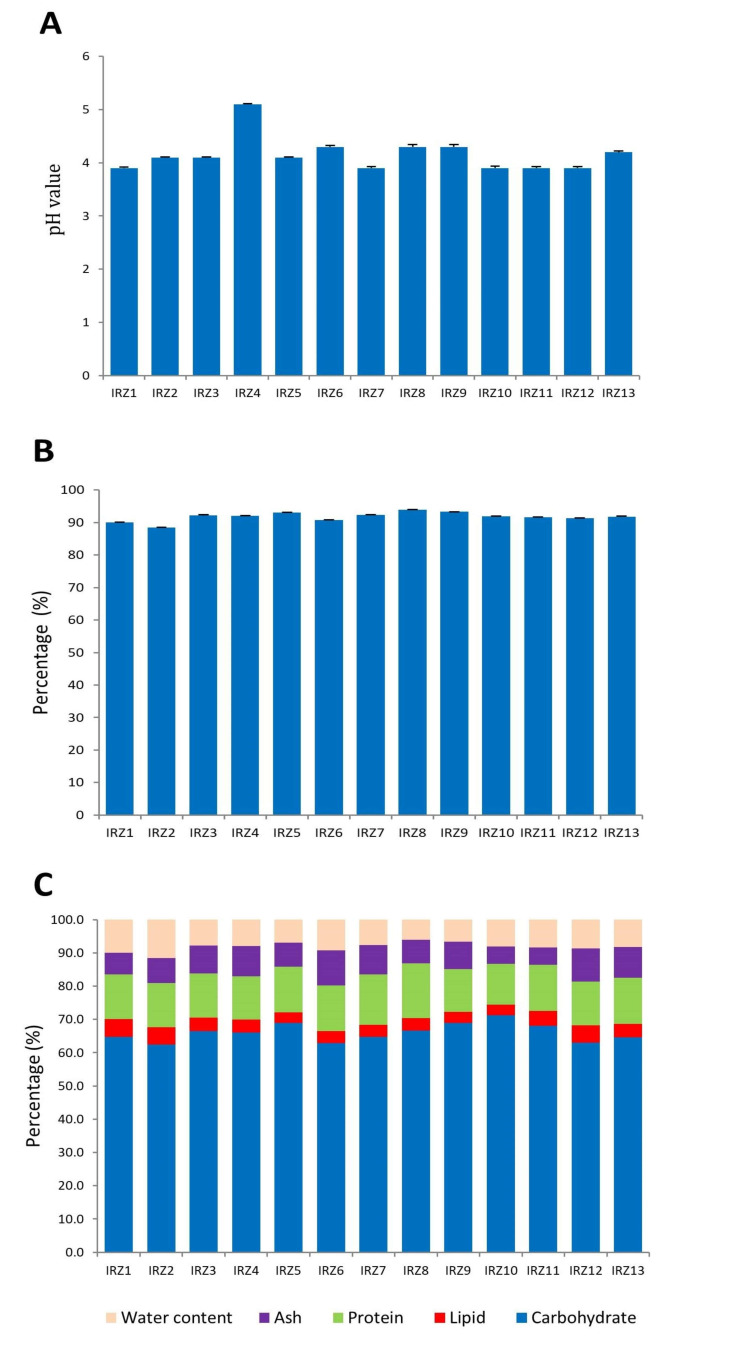
Chemical properties of YC samples: pH (**A**); dry matter (**B**); ash, lipids, protein, and carbohydrates (**C**).

The results of chemical analyses, namely pH, protein and lipid contents, and ash and dry matter weights of YC samples are reported in Figure 5. Regarding pH, all products were acidic due to the fermenting activity carried out by lactic acid bacteria on the milk used to obtain the yogurt that constitutes the basis of this food product. The highest pH (Figure 5A) was recorded for sample IRZ4 (5.1) which was the only sample that was significantly different (*p* < 0.05) from all other samples, which gave values below 4.6 (mean: 4.18). Values below pH 4.6 are sufficient to prevent the growth of most pathogenic bacteria [21], thus enhancing the microbiological safety and shelf life of this product.

Regarding dry matter, which represents the weight of a food product from which water and volatile substances have been removed [22,23], the average percentage considering all YC samples was 91.82%, with values ranging from 88.5% in sample IRZ2 to 93.9% in sample IRZ8 (Figure 5B). These very high values are related to the nature of YC, which is substantially a dried product. This makes YC a product that is very different from most milk-based fermented foods which have a much lower dry matter content, such as yogurt (about 13%).

In Figure 5C, the percentage composition of the main nutrient categories (namely protein, lipids, ash, and carbohydrates) is reported. While the first three components were determined analytically, carbohydrates was determined by subtraction of these three from the dry matter and it comprises all forms of carbohydrates, including monosaccharides, oligosaccharides, polysaccharides, both soluble and insoluble fiber (i.e., starch and cellulose). As shown in Figure 5C, all samples display a similar composition, demonstrating a homogenous manufacturing process adopted among the sampled locations.

### 3.3. Mineral Content of YC Samples

Mean values are 7.91% ± 1.66 for ash, 13.71% ± 1.07 for protein, 4.09% ± 0.73 for lipids and 61% ± 2.13 for carbohydrates.

Ash includes the total amount of minerals in the product that play a significant role in nutritional value, quality, and microbiological stability [24].

Lipid content is important in the dairy industry since it is a key source of energy and fatty acids. Lipids can be also very beneficial at delivering fat-soluble vitamins, such as vitamin A, D, E, and K [25]. Protein plays a key role in the texture and sensory properties of dairy products [25], as well as nutritional and health values [26].

Compared to a similar Iranian dried fermented milk product “dried kashk” [27], YC contains three times less protein and about 1.5 times less fat, while it is more abundant in carbohydrates due to the addition of wheat flour during YC production.

YC is consumed as a soup, which is obtained by dissolving about 6 g of powder in 1 L of water. Considering an average serving of about 250 mL (one dish), the contribution of each serving corresponds to about 0.2 g of protein, 0.06 g of lipid, 0.92 g of carbohydrate and 0.12 g of ash (minerals).

Compared to yogurt, the most diffuse fermented milk, YC provides about four times more protein, the same amount of lipids, 14 times more carbohydrates and 11 times more ash (i.e., minerals). Indeed, due to the dilution of the powder in the soup, the actual intakes are lower.

The mineral content of foods is important in relation to their nutritional value. In the case of YC, this property relies mostly on the characteristics of the milk used to produce the yogurt, which can be related to several factors such as the animal’s genetic background, the type of pasture, and the environmental conditions. The mineral content is also dependent on the other ingredients added during the manufacturing of YC, such as the wheat flour, seasonings and salt. We measured the concentration of seven of the most nutritionally relevant minerals, namely calcium (Ca), potassium (K), magnesium (Mg), manganese (Mn), sodium (Na), phosphorous (P), and zinc (Zn) ions in all YC samples (Table 1). The concentrations of K (mean value: 8038.4 ± 766.1 mg/kg), Mg (mean value: 1655.8 ± 114.0 mg/kg), Mn (mean value: 28.8 ± 3.7 mg/kg), P (mean value: 4059.6 ± 356.4 mg/kg) and Zn (mean value: 27.6 ± 3.3 mg/kg) showed little variation among samples, up to about 10% from the respective means, indicating a quite homogeneous YC composition throughout the regions sampled.

Regarding Ca, its variability appears much higher, probably due to the characteristics of the milk used [28], which represents a major source for this mineral in YC.

Na was present in high amounts (mean: 19.16 g/kg) and its variability was high among the samples, from about 10 to 30 g/kg. Although these values are considerably higher than values reported in the literature regarding other types of “wet” curds [29,30], another Iranian type of dried curd [27] was also found to contain between 38 and 40 g/kg of NaCl. Due to the lack of a standard recipe, different homemade processes can make use of various amounts of sodium chloride, with the aim of influencing both sensory and safety aspects of YC.

By comparing the average mineral content of yogurt with that of YC, many relevant differences can be detected. Apart from the calcium content (1200 mg/kg in yogurt), which is similar on average to that of YC, all other minerals tested are much more present in YC than they are in yogurt. In detail, K is about five times higher, Mg is 13 times higher, Mn is 720 times higher, and P and Zn are about four times higher. The sodium content of YC is 43 times greater than that of yogurt, but this is not surprising since in yogurt there is no addition of NaCl, which is different from YC. Indeed, due to the dilution of the powder in the soup, the actual intakes are lower.

### 3.4. Sugar Content

**Figure 6:**
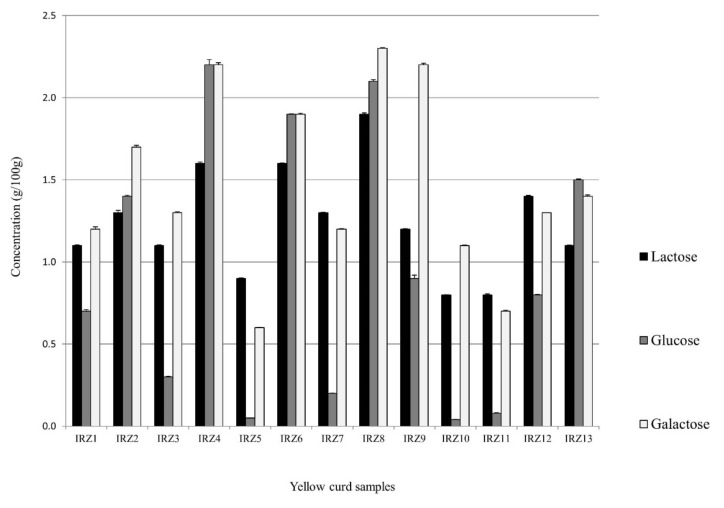
Concentration of sugars in YC samples determined by HPLC analysis. Results are expressed as means ± SD (*n* = 3).

We measured the three quantitatively most important sugars in YC, namely lactose, galactose, and glucose. Lactose comes from milk, where it is normally present at a concentration of 3.5–4%, while galactose is excreted from cells of the LAB strains that are unable to utilize it after lactose hydrolysis. The concentration of galactose in YC samples should be taken into consideration with respect to galactose intolerant people. Many people around the world suffer from galactosemia, which is a sort of disorder caused by the deficiency of galactose-1-phosphate uridylyltransferase (GALT) [31]. This enzyme is important in the Leloir pathway of galactose metabolism [32]. Although morbidity and mortality from galactosemia have been prevented in many countries because of medical screening, in poor districts such as Sistan and Baluchestan provinces, the prevalence could still be high [31]. Glucose comes from plant material, particularly from wheat flour after partial starch hydrolysis. The average concentration of lactose, glucose, and galactose among all samples was 1.28 ± 0.32, 0.98 ± 0.80 and 1.51 ± 0.55 g/100g, respectively (Figure 6). Since the average carbohydrate content is quite similar among the YC samples, the differences in carbohydrate content recorded are likely to be due to microbial activity during fermentation.

Considering the three most abundant LAB genera found in YC samples, all streptococci of dairy origin are able to utilize lactose, whereas only some species of *Lactobacillus* can degrade it, and lactose is normally not metabolized by pediococci [33,34]. Among the lactose-utilizing strains, the capability to use galactose is strain dependent [35]. Glucose is metabolized by pediococci and by lactobacilli, while streptococci use it slowly or not at all, depending on the strain [35,36]. By comparing carbohydrate values with microbiome analysis, we were not able to find any significant correlation with a bacterial category. Therefore, the differences in the carbohydrate pattern are most likely due to the presence of different strains with different metabolic capabilities regarding sugar metabolism. Such differences appear to be quite relevant, particularly in the case of glucose.

## 4. Conclusions

This paper represents the first study on yellow curd, a widespread fermented food largely consumed in some regions of Iran. By using both a canonical and a molecular approach, we were able to characterize the microbiota of YC. In particular, we detected that most bacteria were not alive in the final product, which is stored as a powder, with the exception of a considerable number of putative *Bacillus* spores. In addition, by 16s RNA sequencing, we were able to determine the bacterial categories that developed during the fermentation process. These bacteria, although no longer alive, contributed to define the characteristics of the products and its safety, and they possibly enrich it by producing valuable compounds. This latter possibility was not investigated here and could be a valuable subject matter for further studies. We also measured the main nutritional components and found that YC powder has a good content of protein and lipids, and is rich in carbohydrates (mainly starch and cellulose from plant material and from wheat flour). Unfortunately, the dilution of the powder used to prepare the soup, which represents the most common way of consumption, considerably reduced the nutritional contribution. In this sense, an interesting future development could be the use of the YC powder as base for the preparation of novel functional foods.

## Figures and Tables

**Table 1:** Mineral content of YC samples (mg/kg dry weight). Results are expressed as means ± standard deviation (SD) (*n* = 3).

	Ca	K	Mg	Mn	Na	P	Zn
IRZ1	2414.5 ± 78.5	8062.3 ± 129.2	1706 ± 8.3	27.6 ± 0.1	14,277.9 ± 150.5	3544.4 ± 27.4	27.4 ± 0.1
IRZ2	1627.1 ± 42	9082.2 ± 219.7	1824.1 ± 49.5	33.6 ± 1	18,235.1 ± 569.1	3938.2 ± 147.1	26.7 ± 0.5
IRZ3	2256.9 ± 59.5	7961.4 ± 45.1	1565.8 ± 33.4	22.4 ± 0.2	21,761.1 ± 280.6	3838.9 ± 51.5	25.1 ± 0.4
IRZ4	1709.6 ± 79.3	8581.8 ± 265.8	1690 ± 57.5	30.1 ± 1.4	24,106.6 ± 1051.1	3789.4 ± 153.8	19.6 ± 0.8
IRZ5	1404.6 ± 40.9	7244.9 ± 73.1	1658 ± 12.3	24.7 ± 0.2	17,747.6 ± 187.1	4304.7 ± 38.5	32.7 ± 0.3
IRZ6	1368.4 ± 43.7	7387.5 ± 138.2	1524.7 ± 42.8	27.4 ± 0.4	30,069.1 ± 734.7	3520.3 ± 87.8	32.4 ± 0.7
IRZ7	1965.5 ± 31.3	7675.4 ± 169.5	1594.6 ± 45.9	26.3 ± 0.8	22,176.2 ± 846.1	4500.7 ± 156.1	28.7 ± 0.7
IRZ8	1359.1 ± 13.6	7923.2 ± 41.6	1835.7 ± 6.9	27.8 ± 0.3	15,896.9 ± 67.1	4633.5 ± 34.5	28.7 ± 0.2
IRZ9	2280.1 ± 133.4	8703.7 ± 402.5	1582.6 ± 83.4	25.3 ± 1.4	19,659.3 ± 890.3	4204.5 ± 225.7	26.9 ± 1.5
IRZ10	1476.1 ± 73	7379.1 ± 48.6	1666.8 ± 72.2	33.8 ± 0.7	10,168.6 ± 69.7	4297.6 ± 47.6	24.3 ± 1.1
IRZ11	1860.1 ± 38.9	7554.7 ± 167	1660.1 ± 58.2	30.9 ± 0.7	8911.9 ± 240.4	4400.6 ± 119.7	28.6 ± 0.8
IRZ12	3035.0 ± 107.7	9689.4 ± 280.6	1770.3 ± 42.6	33.7 ± 0.9	23,286.5 ± 376.7	3939.3 ± 92.8	28.2 ± 0.4
IRZ13	1894.7 ± 67.9	7253.5 ± 134.2	1446.4 ± 42.3	31.1 ± 0.9	22,817.1 ± 309.2	3862.7 ± 154.6	29.5 ± 0.7
Mean	1853.1 ± 490.9	8038.4 ± 766.1	1655.8 ± 114.0	28.8 ± 3.7	19,162.6 ± 5637.7	4059.6 ± 356.4	27.6 ± 3.3

## Supplementary Materials

The following are available online at https://www.mdpi.com/2076-2607/8/11/1658/s1, Figure S1: Boxplot of ranked distance obtained in the ANOSIM test between groups and for twelve yellow curd samples obtained from different places in Iran; Figure S2: Plot of the three principal components (PC) determined after principal component analysis (PCA) of 16S rRNA data from twelve yellow curd samples. Technical replicates from the same yellow curd are represented by equal symbols with the same color; and Figure S3: Rarefaction curves generated for each yellow curd sample and their correspondent technical replicates (A, B and C). The number of taxa assigned to OTUs is represented by Taxa S, whereas specimens represent the number of individuals.
