# Supplementary material for: Microbial Diversity and Nutritional Properties of Persian “Yellow Curd” (Kashk zard), a Promising Functional Fermented Food
Source: Microorganisms. 2020 Oct 26;8(11):1658. doi: 10.3390/microorganisms8111658 (PMC7693697; doi:10.3390/microorganisms8111658)
Supplement: Supplementary file 1 [file microorganisms-08-01658-s001.zip › Supplementary Figures.docx]

**Microbial diversity and nutritional properties of Persian *“yellow curd”* (*Kashk zard*), a promising functional fermented food**

Shadi Pakroo, Armin Tarrah, Vinícius da Silva Duarte, Viviana Corich*, Alessio Giacomini

Department of Agronomy Food Natural resources Animal and Environment (DAFNAE), Universityof Padova, viale dell’Università 16, 35020, Legnaro, PD, Italy

*Corresponding author: Viviana Corich ([viviana.corich@unipd.it](mailto:viviana.corich@unipd.it)).

**Supplementary Figure 1**. Boxplot of ranked distance obtained in the ANOSIM test between groups and for twelve yellow curd samples obtained from different places in Iran.


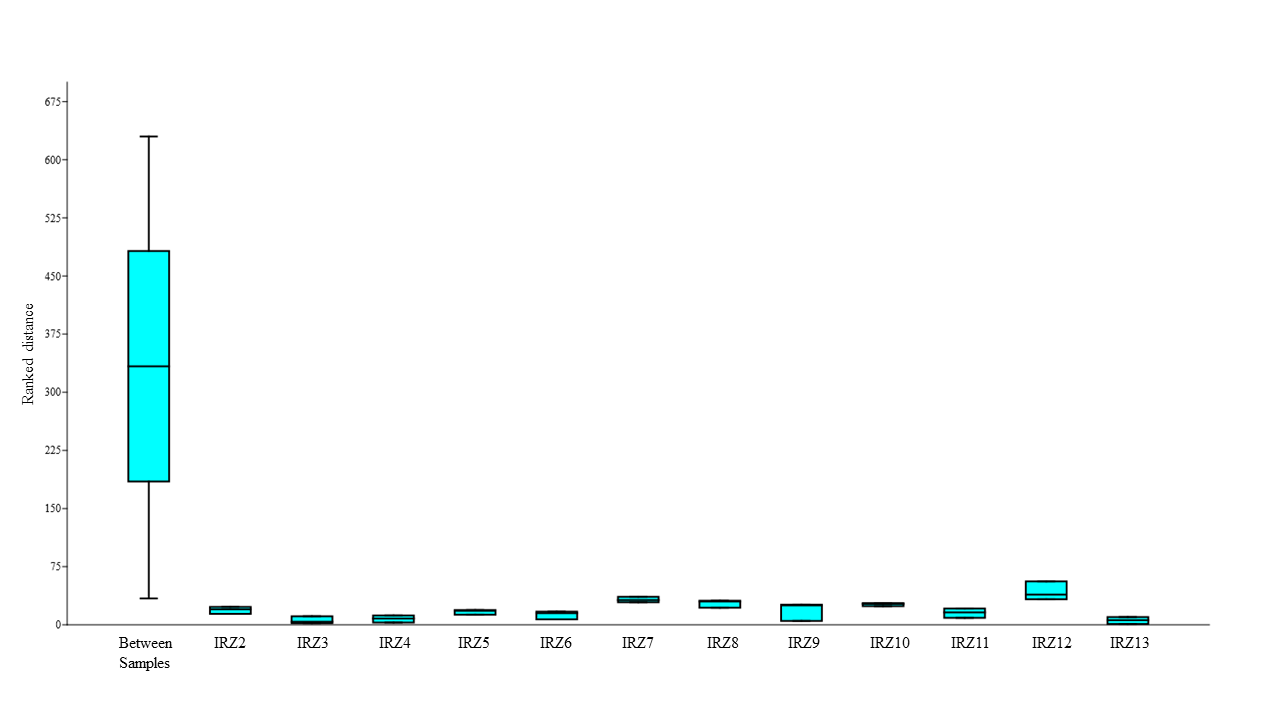


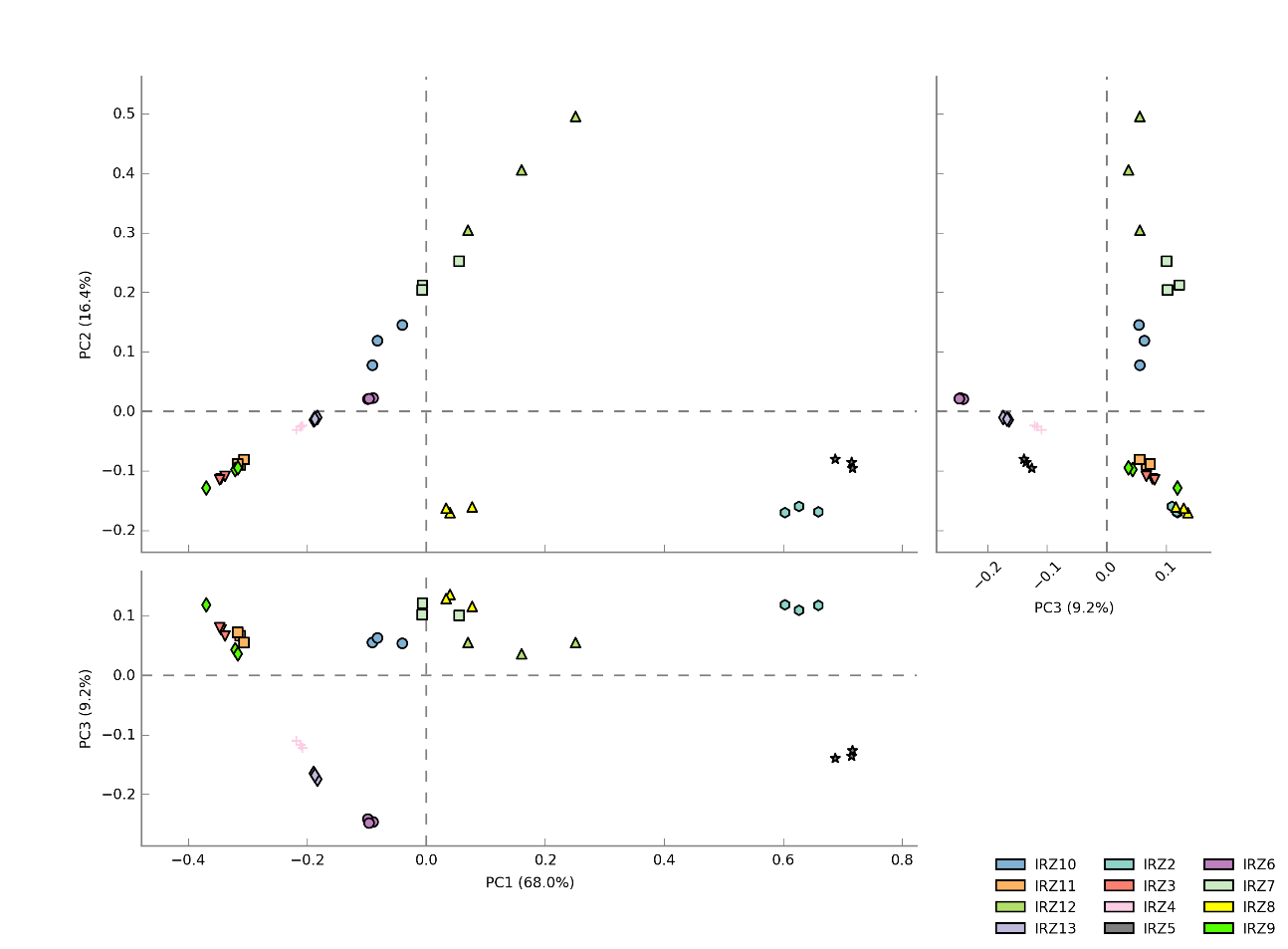


**Supplementary Figure 2**. Plot of the three principal components (PC) determined after principal component analysis (PCA) of 16S rRNA data from twelve yellow curd samples. Technical replicates from the same yellow curd are represented by equal symbols with the same color.


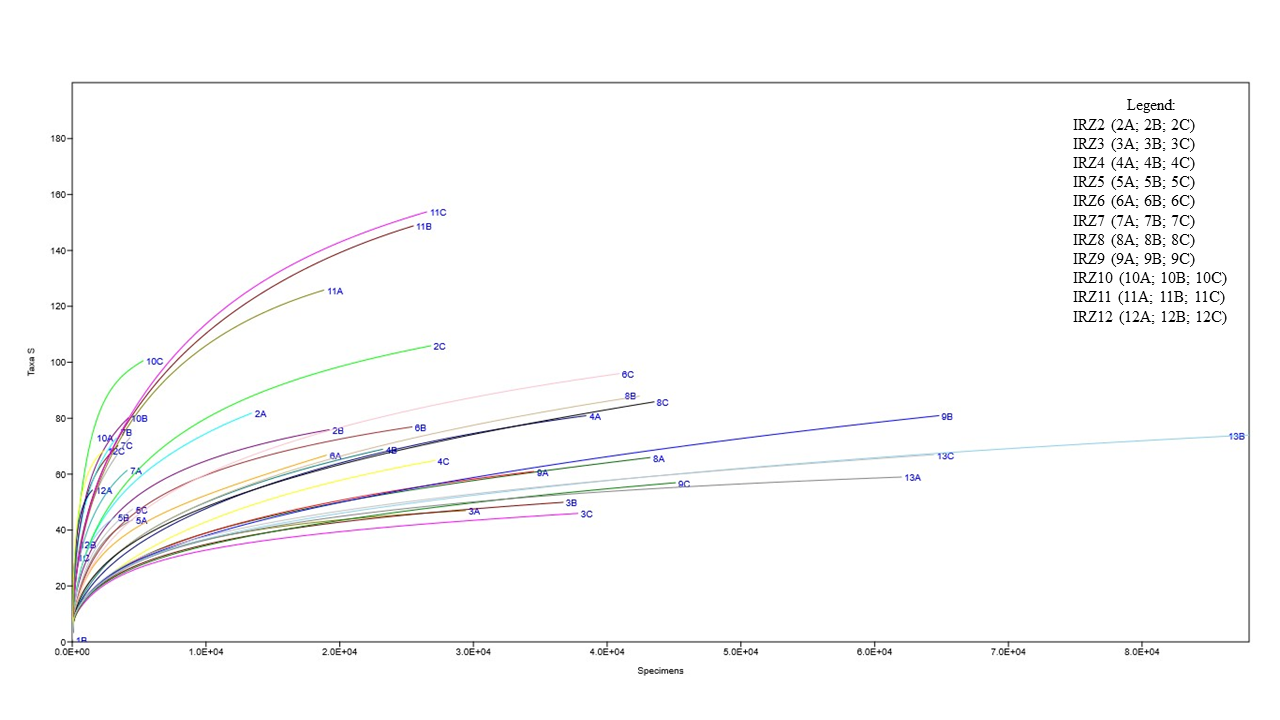


**Supplementary Figure 3**. Rarefaction curves generated for each yellow curd sample and their correspondent technical replicates (A, B and C). The number of taxa assigned to OTUs is represented by Taxa S, whereas specimens represent the number of individuals.
